# Mr. Francis Robert Snuggs Gaman

**Published:** 1910-04-16

**Authors:** 


The death has taken place in London of Mr. Francis Robert Snuggs Gaman, M.R.C.S., L.R.C.P., of Caistor, Lincolnshire, at the age of 40. Mr. Gaman studied at University College Hospital, and gained his diploma in 1894. Later he held the posts of medical officer of health to Caistor and medical officer to Caistor Workhouse.

